# Structural basis of product inhibition by arabinose and xylose of the thermostable GH43 β-1,4-xylosidase from *Geobacillus thermoleovorans* IT-08

**DOI:** 10.1371/journal.pone.0196358

**Published:** 2018-04-26

**Authors:** Ali Rohman, Niels van Oosterwijk, Ni Nyoman Tri Puspaningsih, Bauke W. Dijkstra

**Affiliations:** 1 Department of Chemistry, Faculty of Sciences and Technology, Universitas Airlangga, Surabaya, Indonesia; 2 Institute of Tropical Disease, Universitas Airlangga, Surabaya, Indonesia; 3 Laboratory of Biophysical Chemistry, University of Groningen, Groningen, The Netherlands; Russian Academy of Medical Sciences, RUSSIAN FEDERATION

## Abstract

Complete degradation of the xylan backbone of hemicellulosic plant cell walls requires the synergistic action of *endo*-xylanases and β-1,4-xylosidases. While *endo*-xylanases produce xylooligosaccharides from xylan, β-1,4-xylosidases degrade the xylooligosaccharides into xylose monomers. The glycoside hydrolase family 43 β-1,4-xylosidase from *Geobacillus thermoleovorans* IT-08 is a promising, heat stable catalyst for the saccharification of hemicellulosic material into simple fermentable sugars, but it is competitively inhibited by its products arabinose and xylose. As a first step to help overcome this problem, we elucidated crystal structures of the enzyme in the unliganded form and with bound products, at 1.7–2.0 Å resolution. The structures are very similar to those of other enzymes belonging to glycoside hydrolase family 43. Unexpectedly, the monosaccharides are bound in very different ways. Arabinose preferentially binds in subsite -1, while xylose exclusively interacts with subsite +1. These structures and sugar binding preferences suggest ways for improving the catalytic performance of the enzyme by rational mutational design.

## Introduction

Cellulose, hemicellulose and lignin are the major components of plant cell walls [1]. They are not only cheap and abundant energy stocks, but they also have a high potential to be converted into useful end products [2], such as bioethanol, xylitol, and other simple sugars [3, 4]. In fact, hemicellulose is the second most abundant polysaccharide in nature [5], accounting for approximately one-third of all renewable organic carbon on earth [6].

A major component of hemicellulose is xylan. Xylans are complex heteropolysaccharides consisting of a backbone of β-1,4-linked xylose residues and side chains of arabinose, glucuronic acid or its 4-*O*-methyl-ether. These backbone and side chains can be esterified with acetic, ferulic or *p*-coumaric acids, with varying degree of esterification and nature of the side chains, depending on the source of the xylan [5, 7]. Because of their complex chemical structures, the complete degradation of xylans requires a large variety of cooperatively acting xylanolytic enzymes. Removal of the xylan side chains is catalyzed by α-L-arabinofuranosidases, α-D-glucuronidases, acetylxylan esterases, ferulic acid esterases, and *p*-coumaric acid esterases, while degradation of the xylan backbone requires *endo*-1,4-β-xylanases and β-1,4-xylosidases. *Endo*-1,4-β-xylanase degrades the xylan backbone into β-D-xylopyranosyl oligosaccharides, and, in turn, β-1,4-xylosidase cleaves these xylose oligomers into xylose monomers [8–10].

Xylanolytic enzymes are applied in various industrial processes, such as biopulping of wood, extraction of coffee, maceration of fruits and vegetables, and preparation of high-fiber baking goods [7]. Furthermore, the enzymes hold promise in the saccharification of pretreated agricultural and forestry residues to fermentable sugars [5]. In this process, β-1,4-xylosidase plays a critical role since it cleaves more glycosidic bonds in xylan than any other xylanolytic enzyme [11, 12].

Many of these biotechnological processes require enzymes that are stable at medium to high temperatures. The β-1,4-xylosidase from the thermophilic bacterium *Geobacillus thermoleovorans* IT-08 (Xyl; GenPept accession No. ABC75004; EC 3.2.1.37) was found to be stable up to 55°C. The enzyme has a broad substrate specificity, and is active toward various xylans, xylooligosaccharides, as well as the artificial substrates *p*-nitrophenyl-β-D-xylopyranoside and *p*-nitrophenyl-α-L-arabinofuranoside in the pH range of 5.0–7.5 [13]. These properties make Xyl a promising catalyst for application in the processing of agricultural and forestial residues, and, together with other xylanolytic enzymes, for the saccharification of hemicellulosic material into simple fermentable sugars.

Based on amino-acid sequence similarities to other hemicellulolytic enzymes, Xyl was classified as a member of glycoside hydrolase family 43 (GH43) in the CAZy database (http://www.cazy.org) [14, 15]. Four types of GH43 enzymes are distinguished based on their domain architecture [16]. Xyl is a type II enzyme. All GH43 enzymes hydrolyze glycosidic bonds with inversion of the anomeric configuration *via* a one-step, single-displacement mechanism involving an oxocarbenium ion-like transition state [17–19]. For family GH43 β-1,4-xylosidases, this mechanism involves three key residues, an Asp as general base, a Glu as general acid, and another Asp, which modulates the p*K*_a_ of the general acid and keeps it in the correct orientation with respect to the substrate [17].

Currently, five three-dimensional structures of type II GH43 β-1,4-xylosidases are available in the Protein Data Bank (PDB) database, *i*.*e*. from *Bacillus subtilis* (PDB code 1YIF), *Clostridium acetobutylicum* (PDB 1YI7), *Bacillus halodurans* (PDB 1YRZ), *Geobacillus stearothermophilus* (PDB 2EXH) [17], and *Selenomonas ruminantium* (PDB 3C2U) [20]. These proteins show considerable similarity to each other with amino-acid sequence identities of around 53–71%. In contrast, the sequence of Xyl is quite different from those of the other GH43 β-1,4-xylosidases, with an identity of 35% to the nearest structurally characterized homologue (*G*. *stearothermophilus* β-1,4-xylosidase).

The type II GH43 β-1,4-xylosidase 3D structures show that they all contain a catalytic domain with a five-bladed β-propeller fold [17, 20]. In addition, type II, but not type I, GH43 β-1,4-xylosidases possess a C-terminal β-sandwich domain, which closes off one side of the tube-shaped active site groove to form a pocket. The pocket contains the -1 and +1 substrate-binding subsites, which bind the glycone and aglycone part, respectively, of the substrate’s scissile glycosidic bond [17, 20].

β-1,4-Xylosidases are generally inhibited by their monosaccharide hydrolysis products [5, 11, 21]. In this respect, kinetic studies showed that Xyl is competitively inhibited by arabinose and xylose with *K*_*i*_’s of 6.8 ± 0.6 mM and 76 ± 8.5 mM, respectively [13]. This inhibition reduces the effectiveness of Xyl as a saccharifying agent, since these monosaccharides can easily reach concentrations of about 1 M during the saccharification process [22]. Therefore, we considered that a crystal structure of the enzyme might aid in improving the enzyme for industrial application by mutagenesis. Here, we report three-dimensional structures of Xyl in complex with arabinose, with xylose, and with both sugars together. The structures pinpoint the binding sites of the monosaccharides in Xyl’s active site and portray their binding modes. Although kinetic studies suggested that arabinose can bind in both subsites -1 and +1 of a type II GH43 β-1,4-xylosidase [11, 22], our structural results show that arabinose clearly prefers to bind in subsite -1. In contrast, xylose was found to bind in a single subsite only [11, 22], which we identify as subsite +1; it blocks the active site entrance, thus explaining its inhibitory action. This differential binding interaction has not been observed before and may enable the rational design of Xyl variants that are less prone to product inhibition, and thereby perform better in an industrial context.

## Materials and methods

### Protein preparation

Xyl was overexpressed and purified as previously described [23]. In short, Xyl was overexpressed as a His-tagged protein in *Escherichia coli* BL21 (DE3) from plasmid pET-*xyl* after induction with 1 mM isopropyl-β-D-1-thiogalactopyranoside at 310 K. After cell lysis, the protein was purified by heating to 323 K for 1 h to remove *E*. *coli* proteins, followed by nickel affinity chromatography on a Ni-NTA agarose column (Qiagen), and anion-exchange chromatography on a Resource Q column (Pharmacia Biotech). The purified Xyl was concentrated with a Microsep 10 K Omega concentrator (Pall Corporation) and its concentration was adjusted to 7 mg/ml in 25 mM Tris-HCl, pH 8.0, and 100 mM NaCl. The Xyl stock solution was stored at 253 K.

### Protein crystallization, cryoprotection and ligand soaking

Xyl crystals were obtained at 283 K using the hanging-drop vapor-diffusion method under conditions similar to those previously described [23]. In a typical experiment, 1 μl of Xyl solution was mixed with an equal volume of reservoir solution containing 0.1 M HEPES (*N*-(2-hydroxyethyl)piperazine-*N’*-(2-ethanesulfonic acid)) buffer, pH 7.0, and 5% (*w/v*) PEG 6000. Crystal formation was accelerated by streak seeding using available Xyl crystals as seeds. In these conditions, tetragonal Xyl crystals with dimensions of about 100 x 100 x 100 μm were formed overnight.

Prior to data collection, a Xyl crystal was immersed for 5 s in reservoir solution containing 20% (*v/v*) glycerol before flash-cooling in liquid nitrogen. Xyl crystals with bound L-arabinose (Xyl•arabinose), D-xylose (Xyl•xylose), or L-arabinose and D-xylose (Xyl•arabinose•xylose) were prepared by soaking Xyl crystals for 10 min in reservoir solution supplemented with 500 mM L-arabinose (Sigma-Aldrich), D-xylose (Sigma-Aldrich), or both L-arabinose and D-xylose, respectively. These monosaccharide-bound crystals were cryoprotected in a similar way as for the native crystal with solutions consisting of reservoir solution containing 35% (*v/v*) PEG 300 and the monosaccharide supplements. All cryoprotection and ligand soaking procedures were performed at 283 K.

### Data collection and processing

X-ray diffraction data sets were collected from crystals of Xyl, Xyl•arabinose, Xyl•xylose, and Xyl•arabinose•xylose at 100 K. The data sets of Xyl and Xyl•xylose were obtained at beamline X11 of the EMBL outstation at DESY (Hamburg, Germany), whereas the data sets of Xyl•arabinose and Xyl•arabinose•xylose were obtained at beamline ID14-2 and ID14-1, respectively, of the European Synchrotron Radiation Facility (Grenoble, France). The Xyl crystal belonged to space group *P*4_3_2_1_2, with approximate unit-cell parameters *a* = *b* = 62.1 Å, *c* = 275.8 Å. There is one 61.5 kDa molecule per asymmetric unit, giving a Matthews coefficient of about 2.2 Å^3^/Da, and a solvent content of about 43.1%. The crystals from the soaks had the same space group as Xyl with comparable unit-cell parameters and cell content (Table 1). The data were integrated and scaled with XDS [24] and merged with SCALA [25] from the CCP4 package [26].

**Table 1:** Summary of crystallographic data collection and processing statistics. Values in parenthesis are for the highest-resolution shell.

Data set	Xyl	Xyl•arabinose	Xyl•xylose	Xyl•arabinose• xylose
Beam line	X11/EMBL	ID14-2/ESRF	X11/EMBL	ID14-1/ESRF
Detector	MAR225	ADSC Q4	MAR225	ADSC Q210
Wavelength (Å)	0.9120	0.9330	0.9300	0.9334
Resolution (Å)	46.13–1.70	46.50–2.10	46.35–1.90	46.31–1.70
	(1.79–1.70)	(2.21–2.10)	(2.00–1.90)	(1.79–1.70)
Space group	*P*4_3_2_1_2	*P*4_3_2_1_2	*P*4_3_2_1_2	*P*4_3_2_1_2
Unit cell:				
*a* = *b* (Å)	62.1	60.0	62.5	59.8
*c* (Å)	275.8	279.0	276.7	277.9
α = β = γ (deg)	90.0	90.0	90.0	90.0
Molecules per asymmetric unit	1	1	1	1
Matthew’s coefficient (Å^3^ Da^-1^)	2.16	2.04	2.19	2.02
Solvent content (%)	43.1	39.7	43.9	39.1
*R*_merge_^†^	0.080 (0.386)	0.122 (0.408)	0.086 (0.297)	0.072 (0.273)
*R*_p.i.m._^‡^	0.042 (0.262)	0.049 (0.185)	0.054 (0.199)	0.028 (0.128)
Total observations	249219 (25370)	213731 (23516)	149096 (19109)	428405 (40086)
Unique reflections	60040 (8505)	31118 (4397)	44217 (6152)	57020 (7628)
Mean *I/*σ*I*	12.0 (2.7)	12.0 (3.8)	8.6 (3.4)	17.7 (5.2)
Completeness	99.0 (97.6)	99.9 (99.5)	99.1 (96.7)	99.0 (93.1)
Multiplicity	4.2 (3.0)	6.9 (5.3)	3.4 (3.1)	7.5 (5.3)

^†^ *R*_merge_ = ∑_*h*_∑_*i*_|*I*_*i*_(*h*) − ⟨*I*(*h*)⟩|/∑_*h*_∑_*i*_*I*_*i*_(*h*)

^‡^ *R*_p.i.m._ = ∑_*h*_[1/(*N* − 1)]^1/2^ ∑_*i*_|*I*_*i*_(*h*) − ⟨*I*(*h*)⟩|/∑_*h*_∑_*i*_*I*_*i*_(*h*)

I_i_(h) is the integrated intensity of a reflection, <I(h)> is the mean intensity of multiple corresponding symmetry-related reflections, and N is the multiplicity of the given reflections.

### Structure determination and refinement

Initial phases were obtained from maximum likelihood molecular replacement using the program PHASER [27] within the CCP4 package. The crystal structure of *B*. *subtilis* β-xylosidase (33% identity; PDB 1YIF) was used as starting structure. A clear solution was obtained in space group *P*4_3_2_1_2 with one molecule of Xyl in the asymmetric unit. An initial model was built manually in the electron density map from PHASER, using the visualization program COOT [28]. Alternative positions of side chains of several amino acid residues as well as water and ligand molecules were added to the model with this program, and manually checked and refined against 2*F*_obs_—*F*_calc_ maps. The initial model was then refined with Refmac5 [29] within the CCP4 package. The final model of Xyl was obtained after several rounds of manual building in COOT and refinement in Refmac5. The structures of the monosaccharide-bound Xyls were solved using a similar protocol as for Xyl, with the previously solved Xyl model as the starting structure. *F*obs—*F*_calc_ difference electron density maps revealed additional, monosaccharide-like density in the active site, *i*.*e*. β-L-arabinofuranose and/or α-D-xylopyranose. The coordinates of these monosaccharides were taken from the Refmac5 monomer library. The β-xylosidase coordinates and structure factors have been deposited in the PDB, with structure reference numbers 5Z5D (Xyl), 5Z5F (Xyl•arabinose), 5Z5H (Xyl•xylose), and 5Z5I (Xyl•arabinose• xylose).

## Results and discussion

### Structure analysis

Bipyramid-shaped tetragonal Xyl crystals were obtained from a solution of 5% (*w/v*) PEG 6000 as precipitant in 0.1 M HEPES buffer, pH 7.0, using His-tagged protein (543 amino acid residues with a calculated molecular weight of 61,533 Da), which comprised the complete Xyl protein (511 residues; 57,993 Da), followed by 32 additional residues at the C-terminus (**KGELNSKLEGKPIPNPLLGLDST**RTG **HHHHHH**) containing a V5 epitope and a 6xHis extension (both shown in bold) [23].

X-ray diffraction data sets from Xyl, Xyl•arabinose, Xyl•xylose, and Xyl•arabinose•xylose crystals were collected to resolutions of 1.70, 2.10, 1.90, and 1.70 Å, respectively. The crystals belonged to the primitive tetragonal space group *P*4_3_2_1_2 with a *c* axis of about 280 Å. Crystal parameters, and data collection and processing statistics are presented in Table 1. The crystal structures were solved by molecular replacement using the *B*. *subtilis* β-1,4-xylosidase structure as starting model (33.9% amino acid sequence identity; PDB 1YIF), and refined to the highest possible resolution. Table 2 lists pertinent details of the refinement statistics. The coordinates and structure factors have been deposited in the PDB, with structure reference numbers 5Z5D (Xyl), 5Z5F (Xyl•arabinose), 5Z5H (Xyl•xylose), and 5Z5I (Xyl•arabinose• xylose).

**Table 2:** Refinement statistics.

Crystal	Xyl	Xyl•arabinose	Xyl•xylose	Xyl•arabinose• xylose
Resolution range (Å)	46.13–1.70	45.48–2.10	46.35–1.90	41.85–1.70
Number of reflections	56950	29435	40994	54015
*R-*factor (%)^†^	14.82	14.93	21.61	15.91
*R*_free_ (%)^‡^	17.59	19.95	25.90	19.04
Number of atoms:				
- Totals	4654	4535	4595	4610
- Protein	4053	4053	4053	4053
- Ca^2+^ ion	1	1	1	1
- Water molecules	570	471	531	516
- Glycerol	30	-	-	-
- L-Arabinose	-	10	-	10
- D-Xylose	-	-	10	30
RMSDs:				
- Bond lengths (Å)	0.012	0.012	0.011	0.011
- Bond angles (deg)	1.55	1.54	1.45	1.44
- Chiral volume (Å^3^)	0.10	0.10	0.10	0.10
Ramachandran statistics:^§^				
- In preferred regions (%)	96.35	96.00	96.59	95.94
- In allowed regions (%)	3.04	2.80	2.21	3.25
- In disallowed regions (%)	0.61	1.20	1.20	0.81
Mean *B* values (Å^2^):				
- Overall	17.3	19.6	19.9	16.8
- Protein	15.6	18.7	18.6	15.3
- Ca^2+^ ion	15.3	25.2	18.1	13.3
- Water molecules	29.1	27.6	30.0	28.0
- Glycerol	27.5	-	-	-
- L-Arabinose	-	11.9	-	8.7
- D-Xylose	-	-	25.4	22.1^¶^
PDB code	5Z5D	5Z5F	5Z5H	5Z5I

^†^ *R-*factor = ∑_*hkl*_ ||*F*_P(obs)_| − |*F*_P(calc)_||/∑_*hkl*_|*F*_P(obs)_|, where |*F*_P(obs)_| and |*F*_P(calc)_| are observed and calculated structure factor amplitudes, respectively.

^‡^ *R*_free_ is *R-*factor for 5% of the data not included in the refinement selected in thin shells.

^§^ Ramachandran statistics were obtained from the program COOT [28].

^¶^ *B* value of D-xylose in subsite +1 (see text). Mean *B* value for all three D-xylose molecules is 37.7 Å^2^.

In all refined structures, the electron density maps were clear enough for assigning the positions of amino acid residues 1–304 and 311–510. However, for residues 305–310 and the 33 residues beyond 510, the electron density was too weak to assign their positions with confidence, indicating conformational flexibility and/or crystallographic disorder. Therefore, these residues were not included in the final models. Residues 305–310 are at an inter-domain loop, while the 33 residues beyond 510 are at the C-terminus, and include the 32 residues containing the V5 epitope and the 6xHis extension. All flexible/disordered residues are at the surface of the Xyl molecule and are solvent exposed. In the Xyl structure, 7 amino acid side chains (Ser-4, Ser-22, Asn-49, Met-100, Asp-293, Val-426, and Ser-441) were modeled in two alternate conformations. Most of these side chains have also alternate conformations in the other Xyl structures presented here, although in some cases the density was not clear enough to support a double conformation.

In total 570, 471, 531, and 516 water molecules were identified and refined in the final models of Xyl, Xyl•arabinose, Xyl•xylose, and Xyl•arabinose•xylose, respectively. Most of the water molecules occupy similar positions in all four structures. One calcium ion was defined in each final model on the basis of its electron density, coordination geometry (pentagonal bipyramid) and positional similarity to other GH43 protein structures. Additional electron density in the Xyl structure suggested the presence of five independent glycerol molecules, of which two were in the active site. Similarly, density in the active site of the Xyl•arabinose, Xyl•xylose, and Xyl•arabinose•xylose structures was interpreted as β-L-arabinofuranose, α-D-xylopyranose, and β-L-arabinofuranose and α-D-xylopyranose, respectively. In addition, beyond the active site, two β-D-xylopyranose molecules could be modeled in the Xyl•arabinose•xylose structure. However, these latter binding modes are not specific, since they were absent at the corresponding positions in the Xyl•xylose structure.

A conformational analysis of the polypeptide chains showed that most of the residues in the four structures reported here fall into the allowed regions of the Ramachandran plot, with at least 95.9% of the residues present in the preferred regions. The average overall *B*-factors were approximately 17.3, 19.6, 19.9, and 16.8 Å^2^ for Xyl, Xyl•arabinose, Xyl•xylose and Xyl•arabinose•xylose structures, respectively. Of the five glycerol molecules in the Xyl structure, the one in subsite -1 exhibited the lowest average *B*-factor (10.4 Å^2^). Likewise, the average *B*-factor of β-L-arabinofuranose, in subsite -1 of the Xyl•arabinose and Xyl•arabinose•xylose structures, was about twofold lower than that of α-D-xylopyranose in subsite +1 of Xyl•xylose and Xyl•arabinose•xylose structures. This suggests that subsite -1 has a higher affinity for hydroxyl compounds than subsite +1.

### Overall fold, quaternary structure and domains

The overall folds of the four Xyl models presented here are essentially the same with RMSDs of the 504 common Cα atoms of approximately 0.28, 0.12, and 0.27 Å for, respectively, Xyl•arabinose, Xyl•xylose, and Xyl•arabinose•xylose structures compared to the structure of Xyl. All crystals contain one protein molecule in the asymmetric unit, with dimensions of ~52 x 53 x 82 Å.

A structural similarity search using the DALI server [30] suggested that the structure of Xyl is closely related to the five type II GH43 β-1,4-xylosidase structures in the PDB, *i*.*e*. β-1,4-xylosidases from *B*. *subtilis* (PDB 1YIF), *C*. *acetobutylicum* (PDB 1YI7), *B*. *halodurans* (PDB 1YRZ), *G*. *stearothermophilus* (PDB 2EXH) [17], and *S*. *ruminantium* (PDB 3C2U) [20]. Although the amino acid sequence of Xyl is only 33.9–35.2% identical to sequences of the β-1,4-xylosidases in the PDB, its 3D structure is very similar with overall RMSDs of ~1.3 Å (Table 3).

**Table 3:** Comparison of GH43 family β-1,4-xylosidases.

β-1,4-Xylosidase^†^	Oligomeric state in asymmetric unit	RMSD (Å) ^‡^	Sequence identity to Xyl (%)^§^		
			Full length	N-terminal domain	C-terminal domain
Gthe	1	0.290	100.0	100.0	100.0
Gste	4	1.262	35.2	40.5	27.3
Cace	4	1.345	35.0	39.8	28.8
Bhal	2	1.267	34.8	40.7	25.9
Srum	4	1.279	34.6	38.5	28.7
Bsub	4	1.305	33.9	41.8	22.9

^†^ The β-1,4-xylosidases are from *G*. *thermoleovorans* IT-08 (Gthe; GenPept ABC75004; Xyl), *G*. *stearothermophilus* T-6 (Gste; GenPept AA98625; PDB 2EXH), *C*. *acetobutylicum* ATCC 824 (Cace; GenPept AAK81382; PDB 1YI7), *B*. *halodurans* C-125 (Bhal; GenPept BAB07402; PDB 1YRZ), *S*. *ruminantium* GA192 (Srum; GenPept AAB97967; PDB 3C2U), and *B*. *subtilis* subsp. subtilis str. 168 (Bsub; GenPept AAB41091; PDB 1YIF).

^‡^ Root-mean-square deviations between Cα atoms of pairwise structural alignments of the β-1,4-xylosidases with the Xyl structure as template. The RMSD for Gthe β-1,4-xylosidase is the average RMSD between the Xyl•arabinose, Xyl•xylose, and Xyl•arabinose•xylose structures; for the other enzymes, the RMSD is the average RMSD of the superposition with all available sub-unit structures. The structural alignments were performed using the Secondary Structure Matching (SSM) server [31].

^§^ The N-terminal domain contains the amino acid residues equivalent to residues 1–304 of Xyl, while the C-terminal domain contains the residues that correspond to residues 310–511 of Xyl.

Of the five type II GH43 β-1,4-xylosidases present in the PDB, four crystallized to contain four molecules in the crystallographic asymmetric unit, *i*.*e*. PDBs 1YIF, 1YI7, 2EXH, and 3C2U, whereas one protein crystallized with two molecules in the asymmetric unit, *i*.*e*. 1YRZ (Table 3). Analysis of the crystal structures and the size of their solvent accessible surface areas suggested that the enzymes are present as tetramers in solution [17, 20]. This tetrameric structure displays D2 point symmetry, and is built up of two tight dimers with their dimer two-fold axes perpendicular to each other. Each tight dimer consists of two monomers that are aligned antiparallel to each other, in which the N-terminal β-propeller domain closely interacts with the C-terminal β-sandwich domain of its counterpart and *vice versa* [17, 20]. While most residues used for the dimer interactions are conserved among these enzymes, those involved in stabilizing the tetramer are relatively variable [17, 20]. Indeed, *S*. *ruminantium* β-1,4-xylosidase is present in solution as a mixture of both tetramer and dimer. Although the tetrameric species is dominant in solution, both forms are equally active [20].

Depending on the PEG 6000 concentration, Xyl crystallized either with a single molecule in the crystallographic asymmetric unit (5% *w/v* PEG 6000) or with four molecules (13% *w/v* PEG 6000) [23]. In contrast to all structurally characterized type II GH43 β-1,4-xylosidases, protein assembly analysis using the PDBePISA server [32] suggested that Xyl from both crystal forms is probably stable in solution as a dimer. Yet, most amino acid residues involved in the dimeric interactions of Xyl are different from those used for oligomeric interactions by other β-1,4-xylosidases mentioned above. Unfortunately, experimental verification of the oligomerization state of Xyl in solution has not been possible due to lack of material.

**Fig 1:**
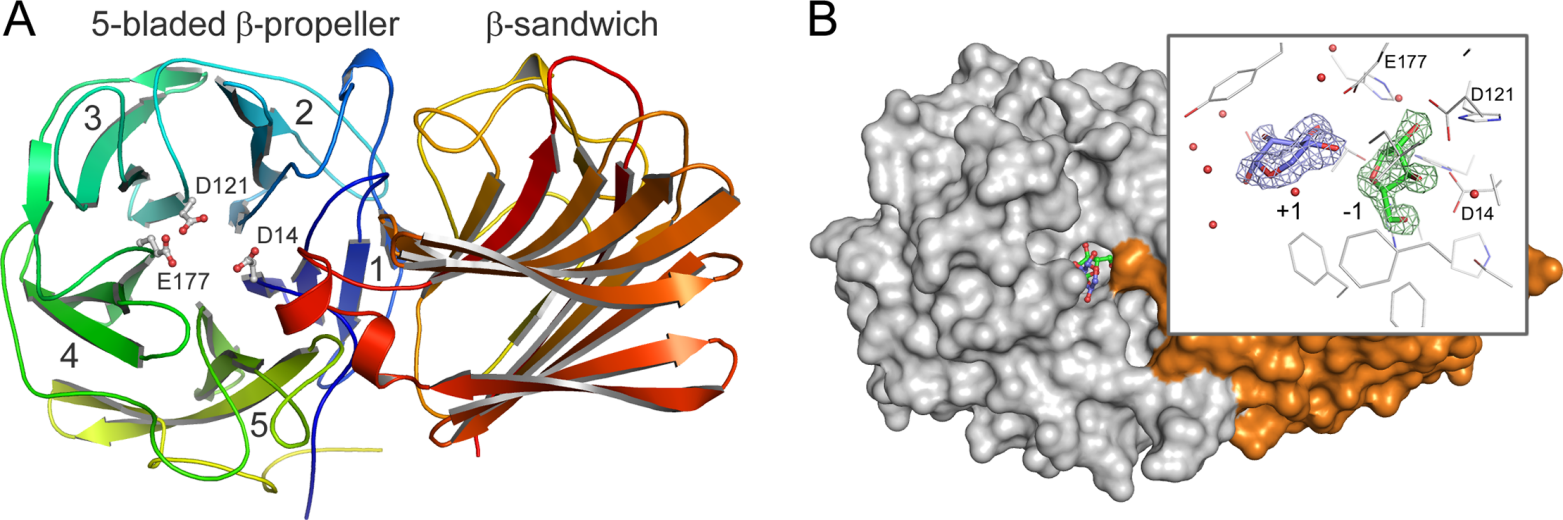
Overall fold of Xyl. A. Ribbon representation of Xyl in rainbow colors from blue (N-terminus) to red (C-terminus). The blades of the N-terminal five-bladed β-propeller catalytic domain are numbered 1–5. The catalytic residues are shown as sticks and colored by element (carbon, gray; oxygen, red). B. Surface representation showing the active site of Xyl with bound β-L-arabinofuranose (carbon atoms in green) and α-D-xylopyranose (carbon atoms in blue) in the Xyl•arabinose•xylose complex structure (PDB 5Z5I). The N- and C-terminal domains are colored in gray and orange, respectively. Inset: The bound monosaccharides in the active site are shown with the *F*_obs_—*F*_calc_ electron density map, obtained prior to refinement, and contoured at 0.5 σ. The subsites of the Xyl’s active site are indicated as -1 and +1. If not specified otherwise, all figures were prepared using the program PyMOL [The PyMOL Molecular Graphics System, v. 0.99, Schroödinger, LLC, http://www.pymol.org].

As observed in other type II GH43 enzymes, Xyl has two domains, an N-terminal five-bladed β-propeller catalytic domain (residues 1–304) and a C-terminal β-sandwich domain (residues 311–511) (Fig 1). These domains are connected by a 6-residue linker (residues 305–310) that is not visible in the crystal structures (see above).

The N-terminal catalytic domain residues form a five-bladed β-propeller (Fig 1A), very similar to that of the catalytic domains of other type II GH43 β-1,4-xylosidases. The β-propeller consists of five β-sheets (1–5 in Fig 1A) in a toroidal arrangement around a central funnel-shaped cavity. Each β-sheet consists of four antiparallel β-strands joined by hairpin turns, with each strand twisted such that the first and fourth β-strands are almost perpendicular to each other. The β-strands at the N-terminal side of the sheets surround the innermost part of the funnel, while the β-strands at the C-terminal side run almost circularly at the outer rim of the funnel. The connections between successive β-sheets are provided by loops from the fourth β-strand of one β-sheet to the first β-strand of the next β-sheet. In contrast, β-sheets 5 and 1 are connected by hydrogen bonds between side chains of the last β-strand of blade 5 and the first β-strand of sheet 1.

The C-terminal residues are less conserved than the residues in the N-terminal domain (Table 3). They fold into a β-sandwich domain similar to the C-terminal domains of the other GH43 β-1,4-xylosidases. The domain consists of two six-stranded antiparallel β-sheets, packed in a face-to-face fashion, which are bent to form a jellyroll-like β-sandwich structure (Fig 1A). Such structures are also observed in the carbohydrate binding modules of many bacterial hemicellulases [33]. A metal ion is bound to the carbonyl oxygen atoms of Asp-316 and Asp-503, and the Ser-344 Oγ and Asp-503 Oδ1 atoms, with three water molecules complementing the coordination sphere. The ion was modeled as a pentagonal-bipyramidal calcium ion, in conformity with the ion observed in other GH43 β-1,4-xylosidases (PDB 1YI7 from *C*. *acetobutylicum* and 2EXH from *G*. *stearothermophilus*). The calcium ion likely has a stabilizing function [34].

**Fig 2:**
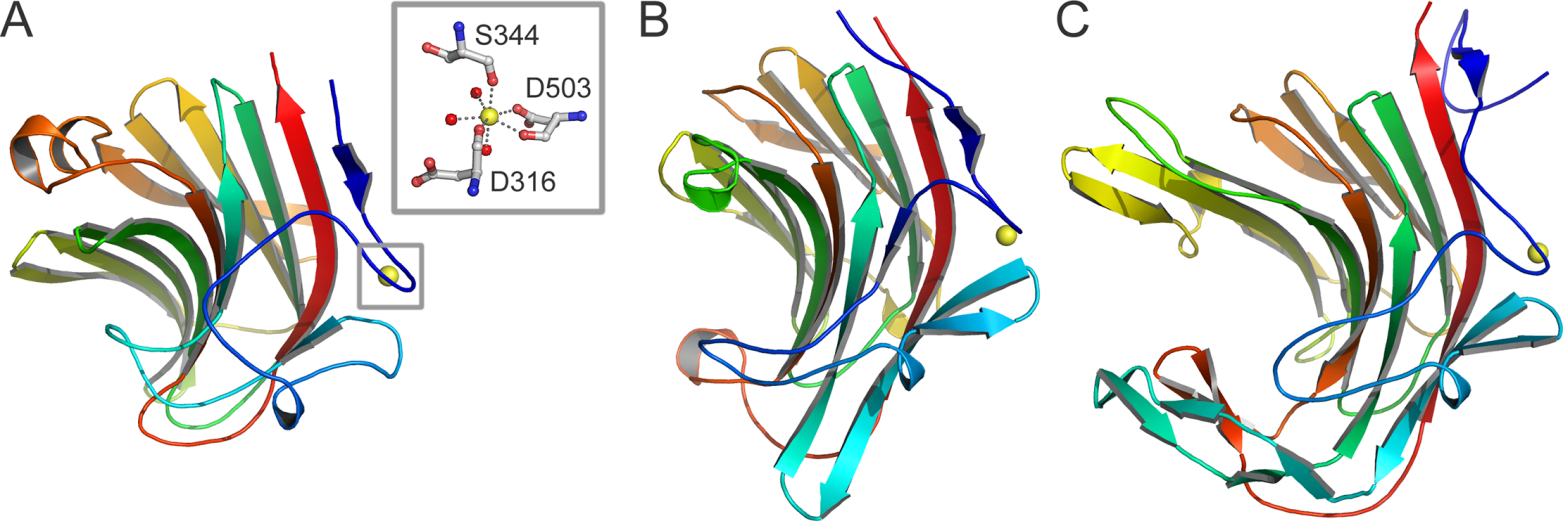
β-Sandwich domain of Xyl in comparison with other enzymes. The C-terminal β-sandwich domain of Xyl (residues 310–511; A) is similar to that of *Bacillus* (1,3–1,4)- β-glucanase H(A16-M) (PDB 1AYH; B) and *P*. *carrageenovora* κ-carrageenase (PDB 1DYP; C). The structures are in about the same orientation and colored in rainbow spectrum from blue at the N-terminus to red at the C-terminus. Inset: Detailed view of the interactions between the C-terminal domain of Xyl and the calcium ion (yellow). A calcium ion has been found at a similar position in the other two enzymes.

Besides its presence in GH43 enzymes, β-sandwich domains are also present in β-glucanases, lectins, cellulases, laminarinases, and sialidases, as revealed by a search with the DALI [30] and PHYRE [35] servers. The highest structural similarity to a non-GH43 enzyme is observed for the hybrid *Bacillus* (1,3–1,4)-β-glucanase H(A16-M) (PDB 1AYH; DALI Z-score 14.8; RMSD of 3.0 Å for 164 Cα atoms) and the κ-carrageenase from *Pseudoalteromonas carrageenovora* (PDB 1DYP; DALI Z-score 14.5; RMSD of 2.8 Å for 167 Cα atoms) (Fig 2). These latter two enzymes show a cleft in their surface, which enables them to bind their carbohydrate substrate. In Xyl the corresponding region is partly filled with loops that probably prevent the binding of ligands. It has been speculated that the C-terminal β-sandwich domain formerly functioned as a carbohydrate-binding module but lost this function during evolution [17].

### Active site

**Fig 3:**
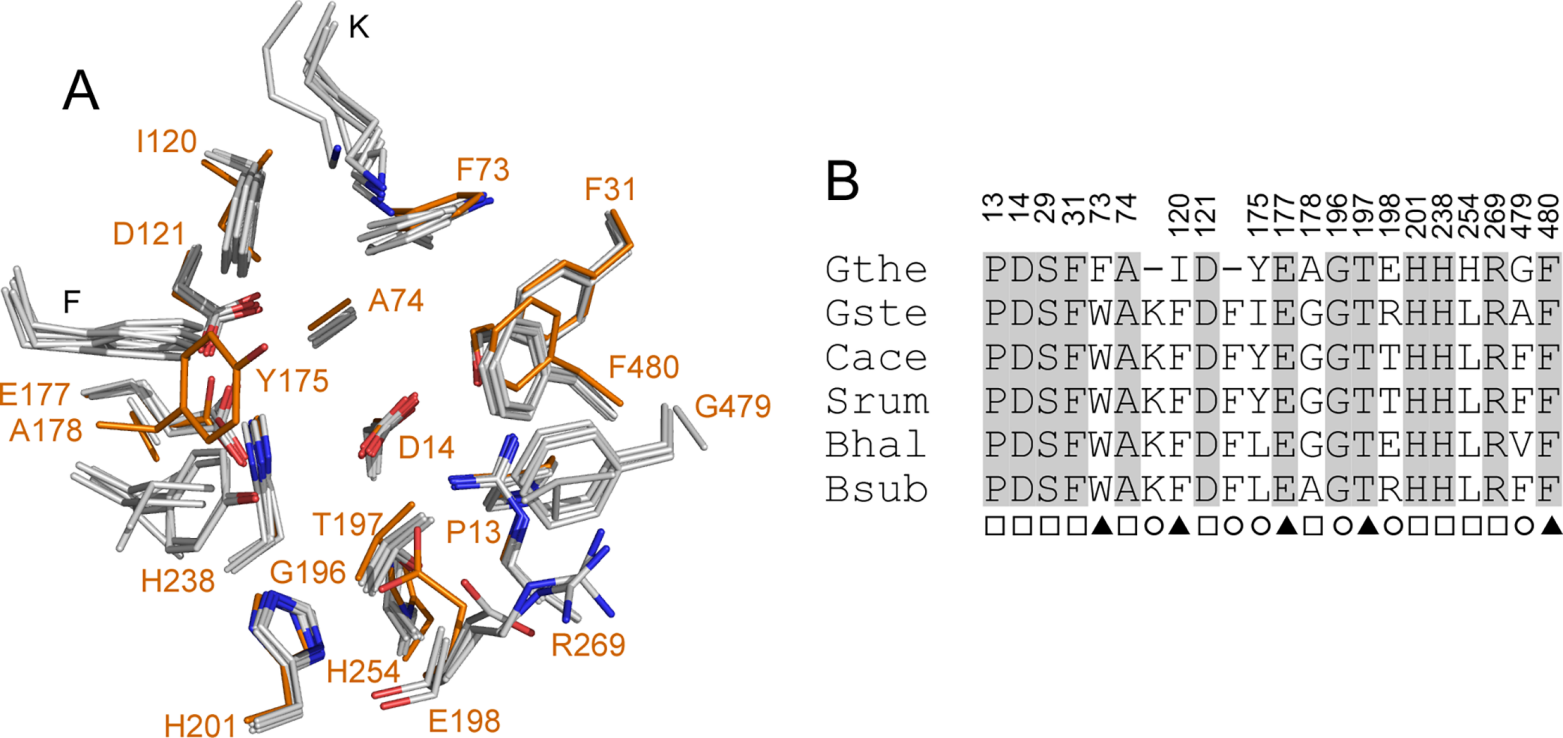
Active site residues in GH43 β-1,4-xylosidases. A. Superposition of the active sites of the β-1,4-xylosidases from *G*. *stearothermophilus* (Gste; PDB 2EXH), *C*. *acetobutylicum* ATCC 824 (Cace; PDB 1YI7), *B*. *halodurans* C-125 (Bhal; PDB 1YRZ), *S*. *ruminantium* GA192 (Srum; PDB 3C2U), and *B*. *subtilis* subsp. subtilis str. 168 (Bsub; PDB 1YIF) onto that of Xyl (Gthe; carbon atoms in orange). Residues are numbered according to Xyl. B. The superposition in A is presented as a sequence alignment and the strictly conserved residues are grey-shaded. The numbering above the alignment refers to Xyl. The marks below the residues indicate their position 5 Å around the subsite -1 (□), +1 (○), or both (▲) of the two-subsite active site of GH43 β-1,4-xylosidases. Fig 3B was prepared manually.

The active site of Xyl is located at the narrow end of the funnel-shaped central cavity of the five-bladed β-propeller domain (Fig 1A). It can accommodate two sugar residues, at subsites -1 and +1 (see below). Substrates can enter the active site pocket only *via* a single route from the solvent (Fig 1B), in agreement with the *exo*-activity of the enzyme, cleaving monosaccharide units from the non-reducing end of the substrate [17, 20]. The active sites of the GH43 β-1,4-xylosidases that are currently available in PDB database are lined with 19 residues from the β-propeller domain and two from the β-sandwich domain (Fig 3). Particularly, the residues in subsite -1 are highly conserved among the enzymes [20]. However, several differences were noted for Xyl. In subsite +1, Phe-73, Ile-120, and His-254 replace Trp, Phe, and Leu present in the five other, structurally characterized GH43 β-1,4-xylosidases. The conserved Lys and Phe (Lys-100 and Phe-155 in *G*. *stearothermophilus* β-1,4-xylosidase) [17] have no equivalent in Xyl. Furthermore, the residues at positions 175, 198, and 479 (Tyr-175, Glu-198, and Gly-479 in Xyl) are variable in the other enzymes. On the other hand, the residues in subsite -1 are fully conserved, including the catalytic base Asp-14, the catalytic acid Glu-177, and Asp-121, which modulates the p*K*_a_ of the catalytic acid and keeps it in the correct, productive orientation.

*Binding of L-arabinose at subsite -1 –*Because in the sugar-free Xyl structure two glycerol molecules from the cryoprotectant were found in the active site (one in each subsite), the carbohydrate-binding studies described below were done in the presence of PEG 300 as cryoprotectant. Sugar-free Xyl crystals cryo-protected with PEG 300 were not stable.

**Fig 4:**
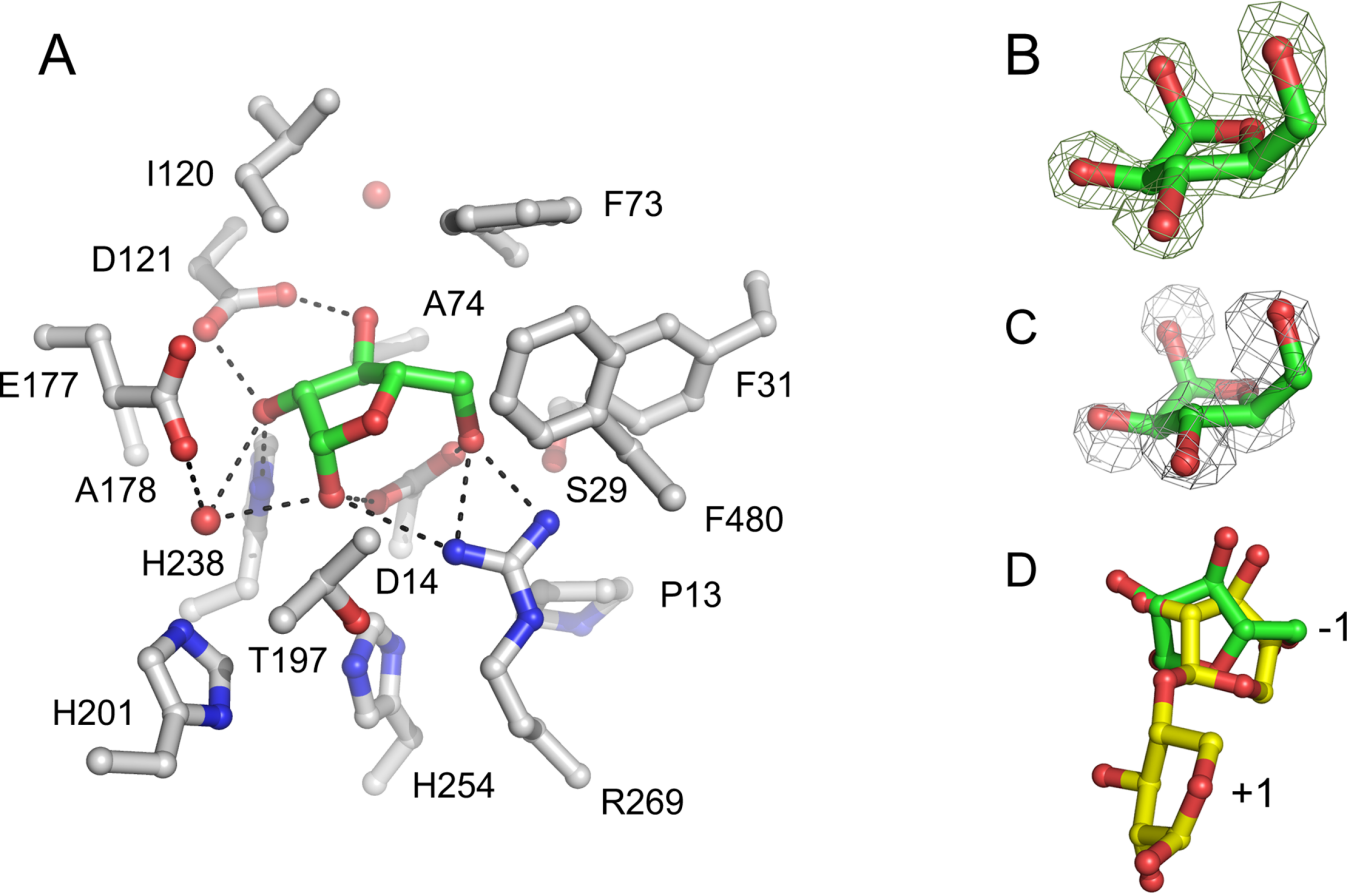
Subsite -1 structure of the active site of Xyl. A. The interactions of β-L-arabinofuranose (carbon atoms in green) with Xyl in subsite -1 of the Xyl•arabinose structure. The amino acid side chains (carbon atoms in gray) and two water molecules (red spheres) within 5.0 Å from the monosaccharide are shown. The protein orientation is approximately the same as in Fig 1. Potential hydrogen bonds between the monosaccharide and active site residues and a water molecule are represented as dashed lines. B. *F*_obs_—*F*_calc_ electron density of bound β-L-arabinofuranose, obtained prior to refinement, contoured at 0.5 σ. C. A superposition of the Xyl•arabinose and Xyl•xylose structures revealed that all arabinose oxygen atoms are at similar positions as water molecules in the Xyl•xylose structure. The 2*F*_obs_—*F*_calc_ electron density map contoured at 1 σ is shown for the five water molecules in subsite -1 of the Xyl•xylose structure. D. A superposition of the active site residues of Xyl and *G*. *stearothermophilus* β-1,4-xylosidase (its active site subsites are indicated as -1 and +1) [17], showing that arabinose (carbon atoms in green) bound in subsite -1 of Xyl is shifted by ~1 Å relative to the xylosyl moiety in that of *G*. *stearothermophilus* β-1,4-xylosidase (yellow).

In both Xyl•arabinose (Fig 4A) and Xyl•arabinose•xylose (Fig 1B) structures, arabinose was only found in subsite -1. Although in subsite +1 of the Xyl•arabinose structure some extra electron density is present, it was not possible to model an arabinose molecule in it, suggesting a weak affinity of the monosaccharide for the subsite, if any. Furthermore, subsite +1 in the Xyl•arabinose•xylose structure is occupied by xylose. This result differs from kinetic studies with *S*. *ruminantium* β-1,4-xylosidase [11, 22] and structural studies with a metagenomic β-xylosidase/α-L-arabinofuranosidase CoXyl43 [36], a type I GH43 enzyme, which suggested that arabinose could bind to both subsites -1 and +1.

In both crystal structures, the arabinose molecule adopts the less stable β-L-arabinofuranose anomer, *i*.*e*. rather than its α-anomer, with *E*_3_ envelope conformation (Fig 4B). Its position is stabilized by hydrogen bonds with Asp-14 (the catalytic base), Asp-121 (the p*K*_a_ modulator), His-238, Arg-269, and, *via* a water molecule, Glu-177 (the catalytic acid). All these amino acid residues are strictly conserved in GH43 β-1,4-xylosidases. In addition, hydrophobic interactions with Phe-31, Phe-73, Ala-74, Ile-120, and Thr-197 contribute to its binding. Phe-31 and Thr-197 are strictly conserved in GH43 β-1,4-xylosidases, and Phe-73 and Ile-120 replace conserved Trp and Phe residues, respectively.

Interestingly, a comparison of the Xyl•arabinose and Xyl•xylose structures revealed that the arabinose hydroxyl groups have very similar positions to the water molecules in the Xyl•xylose structure (Fig 4C). Hydrogen bonds stabilize the axial hydroxyl group configuration at the anomeric C atom of β-L-arabinofuranose. Such stabilization is not possible for α-L-arabinofuranose, in which the anomeric hydroxyl group is in equatorial orientation. This configuration is in agreement with the inverting catalytic mechanism of GH43 β-1,4-xylosidases, which results in the α-anomeric D-xylose product, in which the anomeric hydroxyl group has an axial orientation, similar to the axial orientation of the anomeric hydroxyl group of β-L-arabinose. A water molecule in the Xyl•xylose structure (H_2_O-749 in PDB 5Z5H), at a position corresponding to the axial O1 atom of arabinose in Xyl•arabinose•xylose, is at 2.7 Å from the Oδ1 of the catalytic base Asp-14. Its position suggests that it can be activated by Asp-14 to act as the catalytic nucleophile.

*Binding of D-xylose at subsite +1 –*Xyl has a relatively high *K*_*M*_-value for the hydrolysis of natural substrates (13 mM for xylobiose and 5 mM for xylotriose), probably because of competitive inhibition by the formed product [13, 22]. Several other GH43 β-1,4-xylosidases are also known to have significant affinity for their monosaccharide products [11, 37], and it has been shown that site-directed mutagenesis can be a successful approach to reduce an enzyme’s affinity for D-xylose [38].

**Fig 5:**
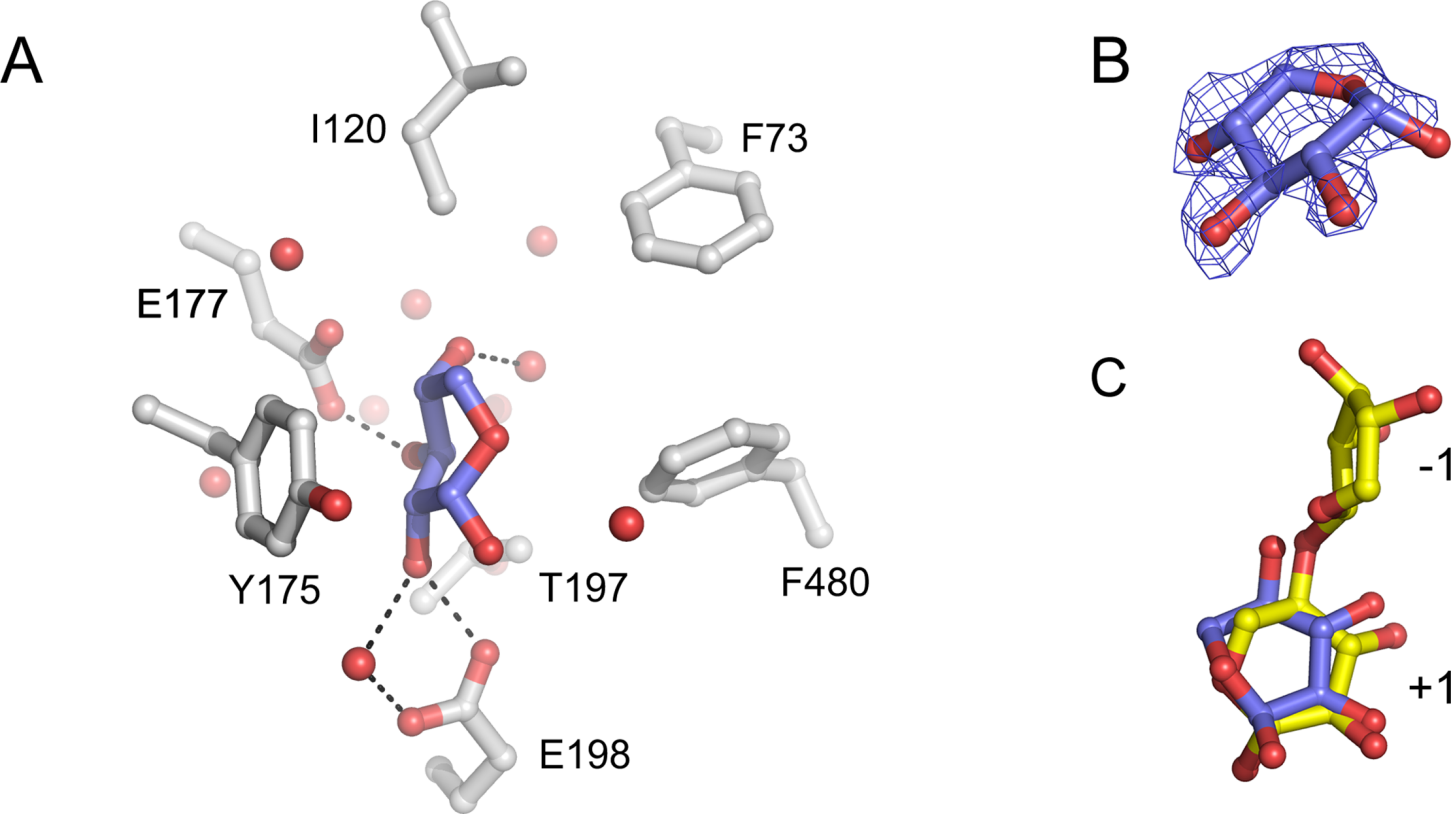
Subsite +1 structure of the active site of Xyl. A. The interactions between α-D-xylopyranose (carbon atoms in blue) and Xyl in subsite +1 of the Xyl•xylose structure. The amino acid side chains (carbon atoms in gray) and water molecules (red spheres) within 5.0 Å from the monosaccharide are shown. The protein orientation is approximately the same as in Fig 1. Potential hydrogen bonds between the monosaccharide and active site residues and water molecules are represented as dashed lines. B. *F*_obs_—*F*_calc_ electron density of bound α-D-xylopyranose, obtained prior to refinement, contoured at 0.5 σ. C. A superposition of the active site residues of Xyl and *G*. *stearothermophilus* β-1,4-xylosidase (its active site subsites are indicated as -1 and +1) [17], revealing that the α-D-xylose molecules in subsite +1 of the two enzymes are bound in a similar orientation.

D-Xylose was reported to bind in the active site of *S*. *ruminantium* β-1,4-xylosidase in one subsite only [11, 22]. Indeed, in the active site of the Xyl•xylose (Fig 5A) and Xyl•arabinose•xylose (Fig 1B) crystal structures, xylose exclusively binds in subsite +1; in subsite -1, water molecules (Xyl•xylose structure; *cf*. Fig 4C) or arabinose (Xyl•arabinose•xylose structure; Fig 1B) are present. This observation is in contrast with the structure of the type I GH43 β-xylosidase/α-L-arabinofuranosidase CoXyl43, in which a xylose molecule was found in subsite -1 of the enzyme [36].

In subsite +1 of Xyl, the D-xylose molecule is bound in the α-D-xylopyranose anomeric configuration, with a ^5^*S*_O_ skew conformation, and thus all hydroxyl groups have equatorial positions (Fig 5B). The molecule is stabilized by hydrophobic interactions with Phe-73, Ile-120, Tyr-175, and Phe-480. Further stabilization is provided by various hydrogen bonds of its O2, O3, and O4 hydroxyl groups. Electron density in subsite +1 of the Xyl•xylose and Xyl•arabinose•xylose structures enabled to model an α-D-xylopyranose molecule in a binding mode similar to that of the xylosyl unit in subsite +1 of a *G*. *stearothermophilus* β-1,4-xylosidase mutant (PDB 2EXJ) [17]. In this binding mode, the O4 hydroxyl group of the xylose points toward subsite -1, while the O1 hydroxyl points away from it. Such a binding mode is supposed to be suitable for catalysis by Xyl. However, a 180^o^-flipped orientation of the xylose, in which the O4 hydroxyl occupies the O1 binding position and *vice versa*, may also be possible, although with somewhat lesser agreement with the electron density. Both binding modes may co-exist, since a xylose monosaccharide, not restrained by a glycosidic bond like in a di-/polysaccharide, may freely rotate or flip.

## Concluding remarks

Complete degradation of xylan requires the synergistic action of *endo*-1,4*-*β-xylanase and β-1,4-xylosidase, as well as arabinofuranosidases and other side chain removing enzymes [8–10]. *Endo*-1,4*-*β-xylanase hydrolyzes the β-1,4 glycosidic bonds in the xylan backbone yielding β-D-xylopyranosyl oligosaccharides, which are further degraded to xylose monomers by β-1,4-xylosidase [39]. However, in the course of the xylan degradation process the released xylose and arabinose monomers may easily accumulate to concentrations well above the *K*_*i*_ [11, 22]. The ensuing inhibition of β-1,4-xylosidase results in the accumulation of xylobiose. Xylobiose in turn, is inhibitory to *endo*-1,4*-*β-xylanase. As a consequence, the overall efficiency of the saccharification process is strongly reduced. Therefore, utilization of a β-1,4-xylosidase with lower affinity for monosaccharides is a desirable objective for the saccharification of xylans.

Here we have shown that arabinose binds in subsite -1 and xylose in subsite +1. Arabinose is mostly stabilized by hydrogen bonds to the protein, and less by hydrophobic interactions, whereas xylose is mostly bound by hydrophobic stacking interactions, and less by hydrogen bonds. The orientation of the arabinose molecule bound in subsite -1 is comparable to that of the xylosyl residue bound in subsite -1 of *G*. *stearothermophilus* β-1,4-xylosidase (Fig 4D) [17]. Except for the O1 hydroxyl group, which points in the opposite direction, all other arabinose hydroxyl groups occupy positions very similar to the xylose hydroxyl groups, albeit shifted by about 1 Å in average. This situation may provide some room for optimization of subsite -1 for binding xylose and lowering its affinity for arabinose, for instance by mutating Arg-269, which has three hydrogen-bonding interactions with the -1 arabinose (Fig 4A), but only two with xylose as observed in the *G*. *stearothermophilus* β-1,4-xylosidase structure [17]. Furthermore, the binding mode of the xylose in subsite +1, either in a binding mode similar to that in a previously published structure (Fig 5C) [17] or in a flipped orientation, reveals two hydrogen bonds, one of them *via* a water molecule, with Glu-198 (Fig 5A). Since this residue is not present in most other type II GH43 β-1,4-xylosidases (Fig 3), it may be a good target for mutation to obtain Xyl variants with lower affinity for xylose. Such experiments are now being initiated.
